# Stratification of Sulfur Species and Microbial Community in Launched Marine Sediment by an Improved Sulfur-Fractionation Method and 16S rRNA Gene Sequencing

**DOI:** 10.1264/jsme2.ME18153

**Published:** 2019-06-11

**Authors:** Hideyuki Ihara, Tomoyuki Hori, Tomo Aoyagi, Hiroki Hosono, Mitsuru Takasaki, Yoko Katayama

**Affiliations:** 1 United Graduate School of Agricultural Science, Tokyo University of Agriculture and Technology 3–5–8 Saiwai-cho, Fuchu, Tokyo 183–8509 Japan; 2 Environmental Management Research Institute, National Institute of Advanced Industrial Science and Technology (AIST) 16–1 Onogawa, Tsukuba, Ibaraki 305–8569 Japan; 3 Institute of Agriculture, Tokyo University of Agriculture and Technology 3–5–8 Saiwai-cho, Fuchu, Tokyo 183–8509 Japan; 4 Department of Food and Environmental Sciences, Faculty of Science and Engineering, Ishinomaki Senshu University 1 Shinmito, Minamisakai, Ishinomaki, Miyagi 986–8580 Japan

**Keywords:** microbial community, sulfur-oxidizing bacteria, sulfur-disproportionating bacteria, launched marine sediment, sulfur fractionation

## Abstract

With a focus on marine sediment launched by the tsunami accompanying the Great East Japan Earthquake, we examined the vertical (*i.e*., depths of 0–2, 2–10, and 10–20 mm) profiles of reduced inorganic sulfur species and microbial community using a newly improved sulfur-fractionation method and 16S rRNA gene sequencing. S^0^ accumulated at the largest quantities at a depth of 2–10 mm, while the reduced forms of sulfur, such as iron(II) sulfide and pyrite, were abundant below 2 mm of the sediment. Operational taxonomic units (OTUs) related to chemolithotrophically sulfur-oxidizing *Sulfurimonas denitrificans* and *Sulfurimonas autotrophica* were only predominant at 2–10 mm, suggesting the involvement of these OTUs in the oxidation of sulfide to S^0^. In addition, *Desulfocapsa sulfexigens*, which is capable of chemolithotrophically disproportionating S^0^, prevailed at the same depth, indicating that accumulated S^0^ was converted to sulfate and sulfide. Although no significant differences were observed in sulfate concentrations across the depths examined, specific species of chemoorganotrophic sulfate reducers, *i.e*., *Desulfotignum toluenicum* and *Desulfosalsimonas propionicica*, showed significantly higher abundance at a depth of 2–10 mm than at the other depths examined. Organic matter potentially generated from sulfur oxidation and disproportionation may have served as the carbon source for the growth of these sulfate reducers. The present results demonstrated that sulfur oxidizers, a sulfur disproportionator, and sulfate reducers played vital roles in sulfur cycling with S^0^ as the key inorganic sulfur species in the oxic-anoxic boundary layer of the launched marine sediment.

On March 11th, 2011, a huge tsunami caused by the Great East Japan Earthquake launched a large amount of marine sediment onto land (24). Due to potentially negative effects on the surrounding ecosystems and human health, geochemical analyses on the launched sediment have been conducted since the disaster (20, 25, 29). Furthermore, microbiological characterization has been performed to obtain a better understanding of the changes that occurred in the geochemical properties of the sediment (2, 3, 13, 16).

Our previous findings indicated that intrinsic microorganisms were responsible for the aerobic and anaerobic transformation of elements in the sediment (2, 13, 16). On-site monitoring and reproductive lab experiments showed that sulfur-oxidizing bacteria (SOB) prevailed in the uppermost layer (depth of 0–2 mm) of the sediment, while the microbial community in the deeper layer (depth of 20–40 mm) did not markedly change with time and sulfate-reducing bacteria (SRB) remained abundant there (16). Since reductants (*e.g*., reduced sulfur compounds) and oxidants (*e.g*., oxygen) are both present in the oxic-anoxic interface just below the uppermost layer, we hypothesized that unique transformations of materials, particularly sulfur compounds, mediated by sediment microorganisms occurred. To gain deeper insights into material flow in the sediment, it is necessary to clarify the vertical profiles of geochemical properties and sulfur-transforming microorganisms.

Inorganic sulfur species serve as the main energy source for SOB and SRB in the sediment. Hsieh and Shieh (15) proposed an analytical method that allows the sequential fractionation of reduced inorganic sulfur species into acid-volatile sulfide (AVS), chromium (II)-reducible sulfide (CRS), and elemental sulfur (ES). Inorganic sulfur species were converted to hydrogen sulfide (H_2_S) by applying different reductants in stages, and the H_2_S produced was then collected in trap solution by diffusion, followed by quantification. In contrast to the distillation method, this diffusion method is capable of fractionation without heating (5, 33). However, it is necessary for the reaction chamber to be open in the diffusion method in order to eject the trap solution at the end of each fractionation stage. Thus, employing sequential fractionation stages within the closed reaction chamber may result in the more convenient handling of the analytical system.

The objective of the present study was to examine the vertical distribution of inorganic sulfur species and microbial community in the launched marine sediment. We herein improved the sulfur-fractionation method by direct flow, not diffusion, of the H_2_S produced into the trap solution. The microbial community structures of different depth layers of the sediment were analyzed by the high-throughput Illumina sequencing of 16S rRNA genes. The microorganisms involved in sulfur cycling of the sediment were identified based on vertical changes in inorganic sulfur species and microbial community.

## Materials and Methods

### Sampling

Details on the sampling site of marine sediment launched by the tsunami accompanying the Great East Japan Earthquake have already been described (16). The on-site sediment that was left untouched on a coastal site at Higashi-matsushima, Miyagi, Japan (38°25′49″N, 141°14′39″E) was collected with a core sampler (60 mm in diameter) on July 2012. The texture of the sediment was solid mud and there was little or no alien material of household goods or plant bodies. The color of the uppermost layer (depth of approximately 0–2 mm) was brown, while that of the deeper layer was black. The collected sediment was transported to the laboratory under cool conditions, divided vertically into depths of 0–2, 2–10, 10–20 and 20–40 mm, and then stored at −80°C until used. Sediment samples from depths of 0–2, 2–10, and 10–20 mm were used for geochemical analyses, the fractionation of reduced inorganic sulfur species, and a microbial community analysis, whereas those from 20–40 mm were only used for the microbial community analysis due to a shortage in the sample amount.

### Geochemical analyses

Ignition loss (IL) of the sediment was measured by drying at 100°C until the weight became constant and heating at 600°C for 2 h. In order to measure the oxidation-reduction potential (ORP) and chloride and sulfate ion concentrations, the sediment was suspended in ultra-pure water at a ratio of 1:2.5 (w/w). After vortexing for 3 min, the ORP of the resultant suspension was measured with an ORP meter (RM-30P; DKK-TOA, Tokyo, Japan). An aliquot of the suspension was diluted appropriately, filtered through a cellulose acetate filter (pore size of 0.2 μm, Advantec, Tokyo, Japan), and then subjected to ion chromatography (883 Basic IC plus; Metrohm Japan, Tokyo, Japan) equipped with a Metrosep A supp 4 column (250×4 mm) and Metrosep A Supp 4/5 guard column (Metrohm Japan) to measure ion concentrations. The remainder of the suspension was freeze-dried and the total amounts of carbon (TC), nitrogen (TN), and sulfur (TS) in the sediment were measured with a CHNS analyzer (FLASH 2000 Organic Elemental analyzer; Thermo Scientific, Waltham, MA, USA).

### Sulfur fractionation and quantification

**Fig. 1:**
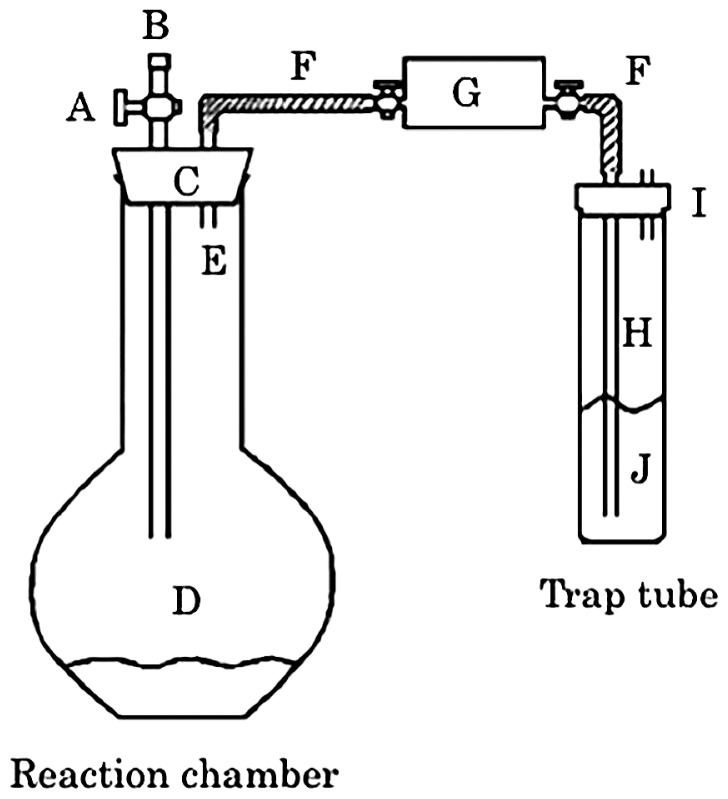
Illustration of improved equipment for the fractionation of sulfur species. A, Glass two-way stopcock; B, double butyl rubber stopper fit on an inlet of A; C, butyl rubber stopper; D, flat-bottomed glass flask (250 mL); E, polypropylene tube; F, polyvinyl chloride (PVC) tube (shaded area); G, polyvinyl fluoride bag (40 to 110 mL) having two-way stopcocks at both ends; H, glass tube, the end of which was immersed not less than 3 cm into the trap solution “J”; I, double butyl rubber stopper fit with a glass tube for the outlet; J, trap solution in a 50-mL glass test tube. Reagents and nitrogen gas were introduced to the equipment through “B” with a syringe and gas cylinder, respectively.

The sulfur-fractionation method described by Hsieh and Shieh (15) was modified for convenience in handling (Fig. 1). Instead of passive diffusion of the H_2_S produced into the trap solution placed in a reaction chamber (14, 15), H_2_S was transferred directly to the trap solution in a glass test tube (Fig. 1J) equipped outside of the reaction chamber (Fig. 1D) by flushing nitrogen gas through an inlet (Fig. 1A). This modification simplified the procedure because it was not necessary to open the chamber in order to replace the trap solution at each fractionation step. Briefly, the sample (wet weight of 10–79 mg) in a 250-mL flat-bottomed glass flask was suspended in 7.5 mL of ultra-pure water. The flask was plugged with a rubber stopper fit with a glass stopcock and polypropylene tube for the inlet and outlet, respectively. The outlet was connected to a polyvinyl chloride (PVC) tube whose end was attached to an adjusted polyvinyl fluoride bag (volume of 40–110 mL, Tedlar bag; Du Pont, Wilmington, DE, USA) carrying two stopcocks for the inlet and outlet. The bag was used to avoid increases in pressure when reagents were added to the flask. The gas phase inside the equipment was replaced with nitrogen gas by flushing at 1 L min^−1^ for 2 min, and the stopcocks at both ends were then closed. To obtain the AVS fraction, 15 mL of deoxygenated 9 M HCl was injected from the inlet into the flask with a syringe and incubated at 30°C for 14 h. The outlet of the bag was connected to a PVC tube and glass tube whose end was dipped in 10 mL alkaline Zn solution (4) in a 50-mL glass test tube. After flushing nitrogen gas for 10 min to trap the evolved H_2_S in the Zn solution, the H_2_S-trapped solution was subjected to iodimetry (4). A total of 10 mM iodine and 10 mM sodium thiosulfate were used for iodimetry. Subsequently, 15 mL of a deoxygenated 2 M Cr(II) solution, which was prepared by dissolving chromium chloride with amalgamated Zn (Zn[Hg]) in 0.5 M HCl (14, 15), was injected with a syringe into the flask used for the quantification of AVS. After being maintained at 30°C for 48 h, volatilized H_2_S was quantified as described above to measure the amount of CRS. Finally, 20 mL of 99.5% *N,N-*dimethylformamide, 5 mL of 2 M Cr(II), and 5 mL of 9 M HCl were injected with syringes into the same flask. After being maintained at 30°C for 24 h, ES was quantified as described above. On the other hand, the recoveries of sulfur from the AVS, CRS, and ES fractions were evaluated using the representative compounds of iron(II) sulfide (FeS; Wako, Osaka, Japan), pyrite (FeS_2_; Alfa Aesar, Haverhill, MA, USA), and sublimed sulfur (S^0^; Wako), respectively. The concentrations of sulfur in FeS and FeS_2_ were measured with the CHNS analyzer. Sublimed sulfur was dispersed in advance according to the method described by Rohwerder and Sand (28) to eliminate large grains of sulfur. The recovery evaluation of the AVS fraction was conducted in quadruplicate and other evaluations and sample analyses were conducted in triplicate.

### High-throughput Illumina sequencing of 16S rRNA genes

The microbial community was analyzed in triplicate by the high-throughput Illumina sequencing of the V4 region of 16S rRNA genes with a MiSeq sequencer (Illumina, Tokyo, Japan) as described previously (2, 13, 16). Briefly, DNA was extracted from the sediment sample in triplicate using the bead-beating method. Each DNA extract was subjected to PCR using the prokaryote universal primer set 515F/806R containing Illumina adapter sequences (7). The reverse primer was encoded with 12-bp barcodes for multiplex sequencing (8). PCR products were purified with an AMPure XP Kit (Beckman Coulter, Brea, CA, USA) and by gel purification. The purified products and an internal control (PhiX Control v3, Illumina) were subjected to paired-end sequencing with a 300-cycle MiSeq Reagent kit (Illumina). The removal of the internal control PhiX and chimeric sequences, filtration by quality value 30 (Q30), and assembling sequences were conducted as described previously (17). A total of 518,073 sequences were obtained from 12 libraries and their average length was 254 bp. The phylogenetic classification of operational taxonomic units (OTUs) with a cut-off value of 97% sequence identity, and the calculation of alpha-diversity indices and a principal coordinate analysis (PCoA) were conducted with the software QIIME ver 1.7.0 (6). Some of the abundant OTUs and OTUs with high increasing ratios were compared to sequences registered in the database of the National Center for Biotechnology Information (NCBI) using the Basic Local Alignment Search Tool (BLAST) to identify their closest cultured relatives (http://blast.ddbj.nig.ac.jp/blastn?lang=ja) (1). The sequence data obtained in the present study have been deposited in the DNA Data Bank of Japan (DDBJ) Sequence Read Archive (DRA) under accession number DRA005893.

## Results

### Geochemical properties of the launched sediment

ORP values in the sediment decreased from 161 to −106 mV with increasing depths (Table 1). The lower ORP value (−58 mV) at 2–10 mm than at 0–2 mm (161 mV) indicated that the oxic-anoxic boundary layer formed at a depth of 2–10 mm of the sediment. IL, TC, and TN values were the highest at 0–2 mm. The concentrations of the chloride ion ranged between 20.0 and 26.2 g kg^−1^ dry weight (dw), showing the high salinities of the sediment. These concentrations were similar, for example, with 10.6–14.2 g L^−1^ in marine pore water and 18.4–19.2 g kg^−1^ in marine sediments reported previously (18, 21). The concentration of the sulfate ion at 0–2 mm was 1.71 gS kg^−1^ dw, which was higher than those (1.24–1.37 gS kg^−1^ dw) at the other depths (Table 2). The TS value in the uppermost layer was 7.25 gS kg^−1^ dw, whereas those below 2 mm of the sediment ranged between 12.04 and 13.49 gS kg^−1^ dw. These results suggest that organic and/or reduced inorganic sulfur species, other than sulfate ions, accumulated below 2 mm of the sediment.

### Inorganic reduced sulfur species revealed by the improved sulfur-fractionation method

To clarify the sulfur species that accumulated below 2 mm of the sediment, reduced inorganic sulfur fractions (AVS, CRS, and ES) were examined using the newly improved sulfur-fractionation method, in which flowing nitrogen gas was employed instead of passive diffusion. To evaluate validity, 0.06–0.47 mgS of FeS, FeS_2_, and S^0^, representative compounds of AVS, CRS, and ES, respectively, were subjected to this method. The results obtained showed that the recovery ratios of the AVS, CRS, and ES fractions were 94.9, 97.8, and 99.9%, respectively (Table S1), which were similar to those using the original method, indicating that our improved method was applicable for detecting slight amounts of reduced inorganic sulfur species.

The concentrations of AVS, CRS, and ES were found to be 2.70–3.09, 6.29–9.38, and 1.84–3.60 gS kg^−1^ dw, respectively, below 2 mm of the sediment (Table 2). These values were markedly higher than those (0.23, 0.33, and 0.37 gS kg^−1^ dw) at 0–2 mm, indicating that cumulated reduced inorganic sulfur species were responsible for the high TS values below 2 mm of the sediment. CRS was the most abundant sulfur fraction at 2–10 and 10–20 mm, suggesting that FeS_2_, a representative compound of CRS (5, 14, 15, 33), is a major sulfur species at these depths. Notably, the concentration of ES at 2–10 mm was twice that at 10–20 mm, whereas no significant differences were observed in the concentrations of AVS and CRS between these depths. The total amounts of measured inorganic sulfur fractions, *i.e*., the sum of AVS, CRS, and ES and sulfate-S, were 2.65, 13.83, and 15.67 gS kg^−1^ dw at depths of 0–2, 2–10, and 10–20 mm, respectively. The amounts at depths of 2–10 and 10–20 mm were similar to or slightly lower than the TS values obtained. These TS values may have been underestimated due to the volatilization of certain sulfur species during freeze-drying. The amount at 0–2 mm was approximately one third of the TS value, implying that the chemical form of most of the sulfur species was not inorganic, but organic in the uppermost layer of the sediment.

### Vertical distribution of sediment microorganisms

**Fig. 2:**
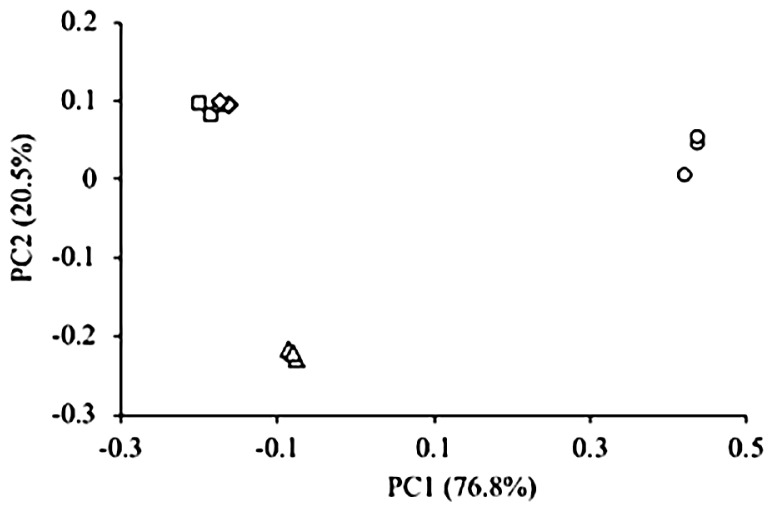
Comparison of microbial community structures in different layers of the sediment based on a Principal Coordinate Analysis (PCoA). These plots were calculated from an equal number of sequences (*n*=37,762) by a weighted UniFrac analysis. ○, 0–2 mm; Δ, 2–10 mm; □, 10–20 mm; ⋄, 20–40 mm.

The microbial community in the sediment sample was characterized by the high-throughput Illumina sequencing of 16S rRNA genes. The total number of sequences obtained from 12 sediment samples was approximately five hundred thousand, corresponding to an average of 43,173 sequences per library (Table S2). The number of microbial OTUs and value of Chao1 indicated that the microbial community at 0–2 mm had the lowest microbial richness. On the other hand, the value of Simpson’s reciprocal at 2–10 mm was less than that at 0–2 mm. Since Simpson’s reciprocal assesses microbial diversity based on microbial richness and evenness, certain microbial species were considered to be predominant at 2–10 mm. A PCoA plot showed distinct microbial communities at depths of 0–2, 2–10, and 10–20 mm (Fig. 2). The microbial community structure at 10–20 mm was similar to that at 20–40 mm, in which anaerobic microorganisms have been reported to prevail (16), suggesting that these layers were anoxic. The oxic-anoxic boundary at 2–10 mm had a unique microbial community from those found in the oxic and anoxic layers.

**Fig. 3:**
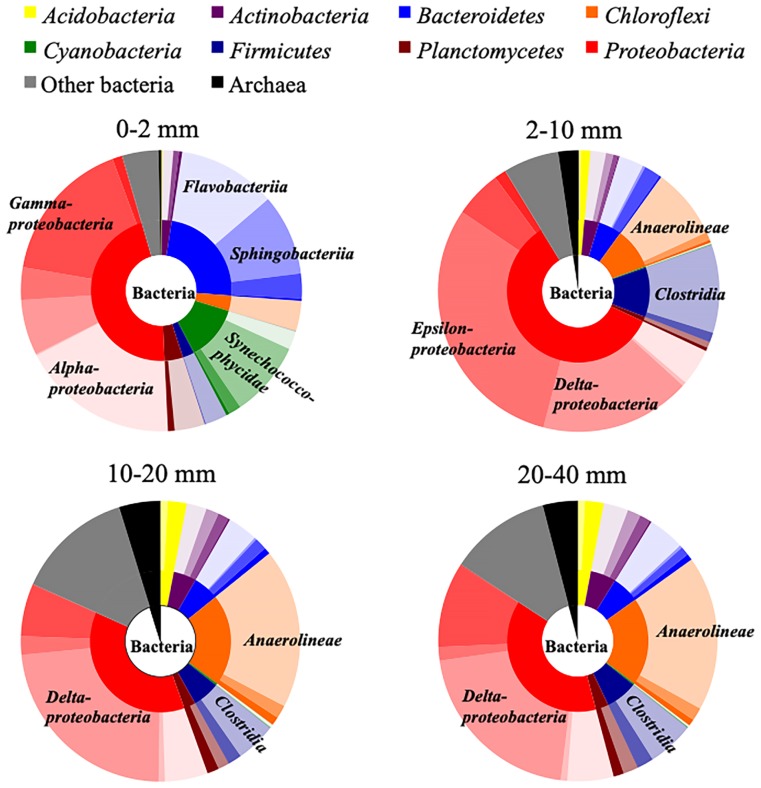
Microbial community structures in different layers of the sediment. Relative abundance was calculated as an average of triplicate measurements. Small, medium, and large circles present the taxa of the domain, phylum, and class (subclass for the phylum *Cyanobacteria*), respectively. Domains and phyla are distinguished by color described in an explanatory note on the upper side. Different classes are shown by color density.

Phylogenetic analyses clearly showed the vertical distribution of microorganisms in the sediment (Fig. 3). At a depth of 2–10 mm, among the most abundant phylum *Proteobacteria* (relative abundance: 59.3%), the class *Epsilonproteobacteria* (30.4%) was predominant and its relative abundance was nearly ten-fold higher than those (1.6–3.7%) in the other layers. The class *Deltaproteobacteria* accounted for 17.3% of the total population. Almost all OTUs in *Epsilonproteobacteria* were assigned to the family *Helicobacteraceae*, whereas those in *Deltaproteobacteria* were assigned to three different families, *i.e*., *Desulfobulbaceae* (9.7%), *Desulfobacteraceae* (3.2%), and *Desulfuromonadaceae* (2.1%) (Fig. S1a). The relative abundance of *Desulfobulbaceae* in the oxic-anoxic boundary layer was higher (*i.e*., 9.7%) than those (5.9–6.7%) in the deeper layers. The second dominant phylum *Firmicutes* (11.3%) mainly consisted of the class *Clostridia* that occupied 10.1% of the total population (Fig. 3). Predominant families in this class were *Clostridiaceae* (4.6%) and *Halanaerobiaceae* (3.8%), and their relative abundance was higher than those (1.4–2.7% and 0–0.9%) in the other layers (Fig. S1b).

At a depth of 2–10 mm, OTU 15133 accounted for 25% of the total population (Table 3 and Fig. S2a), likely resulting in the lowest value of Simpson’s reciprocal (Table S2). This OTU was related to chemolithotrophically sulfur-oxidizing *Sulfurimonas denitrificans* (Accession No.: L40808, sequence identity: 94%). OTU 4080, which was related to some extent to the epsilonproteobacterial sulfur oxidizer *Sulfurimonas autotrophica* (CP002205, 90%), prevailed (2.3%). Bacteria in the genus *Sulfurimonas* are found under sulfidic conditions, including hydrothermal vents, marine sediment, sea water, and terrestrial environments (12). Although these sequence identities with the predominant OTUs were low, the phylogenetic lineages of the relatives suggested that these bacteria oxidized reduced inorganic sulfur species in the oxic-anoxic boundary layer. Following the *S. denitrificans* relative, *Desulfocapsa sulfexigens* (OTU 25758, Y13672, 97%) belonging to *Desulfobulbaceae* of *Deltaproteobacteria* was predominant (7.1%). *Desulfocapsa sulfexigens* is found in the oxic surface of marine sediments and is capable of chemolithotrophically disproportionating S^0^ (10), indicating that S^0^ that accumulated in the layer was most likely used for its growth. Moreover, concerning *Deltaproteobacteria*, chemoorganotrophically sulfate-reducing *Desulfotignum toluenicum* (OTU 20456, MG264282, 100%) and *Desulfosalsimonas propionicica* (OTU 20168, DQ067422, 99%) only proliferated in the oxic-anoxic boundary layer (Table 4). This specific localization was also noted for OTUs 15113 and 7171, which appear to be related to *Alkaliphilus oremlandii* (MG264216, 88%) and *Halanaerobium acetethylicum* (U32594, 88%) (Fig. S2b).

At a depth of 0–2 mm, the phyla *Bacteroidetes* (23.8%) and *Cyanobacteria* (12.6%) were abundant. *Flavobacteriia* (11.3%) and *Sphingobacteriia* (9.3%) were the major classes of *Bacteroidetes*, suggesting that aerobic microorganisms predominated in the uppermost layer. The high relative abundance of the subclass *Synechococcophycidae* (8.7%) within *Cyanobacteria* indicated the proliferation of photolithotrophs. The most abundant OTUs were chemoorganotrophs and photolithotrophs (Table 3). Below 10 mm of the sediment, the phyla *Chloroflexi* (20.1–20.9%) and *Firmicutes* (6.3–7.3%) were abundant, next to *Proteobacteria* (37.2–38.3%). The most predominant classes of *Proteobacteria*, *Chloroflexi*, and *Firmicutes* were *Deltaproteobacteria* (20.8–23.2%), *Anaerolineae* (17.9–18.4%), and *Clostridia* (4.7–5.4%), respectively, indicating that anaerobic microorganisms occupied most of the microbial community. The predominant OTUs 1686 and 18095 were somewhat related to sulfate-reducing *Desulfobulbus elongatus* (Accession No.: X95180, sequence identity: 92%) and *Desulfofaba fastidiosa* (AY268891, 95%), respectively.

## Discussion

By using the combined approach of a newly improved sulfur-fractionation method and the high-throughput Illumina sequencing of 16S rRNA genes, we herein investigated the microbial sulfur cycle in the oxic-anoxic boundary layer of the launched marine sediment. The results of the microbial community analysis suggested that the predominant *S. denitrificans* relative (OTU 15133) and *S. autotrophica* relative (OTU 4080) at 2–10 mm oxidized reduced inorganic sulfur species (Table 3). However, the concentration of the sulfate ion in this layer was only slightly different from that at 10–20 mm (Table 2), implying the limited significance of sulfide oxidation to sulfate. On the other hand, S^0^ clearly accumulated at 2–10 mm (Table 2). The low ORP value (^−^58 mV) at 2–10 mm indicated that the electron acceptor oxygen available for SOB was limited. Some of the cultured SOB may oxidize reduced inorganic sulfur species to S^0^ instead of sulfate if the electron acceptor is not fully available (19, 27). Based on the thermodynamic theory, the oxidation of reduced inorganic sulfur species to S^0^, rather than sulfate, may produce energy efficiently under oxygen-limiting conditions (11). Sulfide quinone oxidoreductases (Sqr), flavocytochrome *c* (Fcc), and the sulfur oxidation complex (Sox) pathway lacking Sox CD have been reported to produce S^0^ as the metabolic intermediate from biochemical aspects (11). The genome of *S. denitrificans* indicated that this bacterium was capable of producing S^0^ via the Sqr and Sox pathways (30). A previous study on microaerophilic wastewater biofilms suggested the contribution of *S. denitrificans* to the accumulation of S^0^ (26). *S. autotrophica* also has the Sqr pathway to potentially produce S^0^ (34). Consequently, bacteria belonging to the dominant OTUs 15133 and 4080 most likely produced S^0^ as the main metabolite of sulfur oxidation in the oxic-anoxic boundary layer. Furthermore, according to our previous findings showing the temporal dynamics of the microbial community in the sediment (16), the *S. denitrificans* relative (OTU 15133) corresponded to a predominant OTU (indicated as OTU 11288 in the reference article [16]) at a depth of 0–2 mm on December 2011, which was seven months before the sampling time of the present study. This finding indicated that the habitat of this bacterium moved with time to a deeper layer, a depth of 2–10 mm, of the sediment. Since the *S. denitrificans* relative may use reduced sulfur compounds for its growth, the shortage of substrates at a depth of 0–2 mm of the sediment led to a decrease in the *S. denitrificans* relative (Table 2 and Fig. S2a). On the other hand, reduced sulfur compounds still remained at a depth of 2–10 mm of the sediment. ORP values suggested that the oxic-anoxic interface was at a depth of 2–10 mm. These environmental conditions were considered to be suitable for the proliferation of the *S. denitrificans* relative. The *S. denitrificans* relative in the sediment may flexibly change the product of sulfur oxidation from sulfate to S^0^ or vice versa in response to redox conditions, as reported previously for sulfur-oxidizing *Thiobacillus thioparus* (32).

It is also important to note that sulfur-disproportionating *Desulfocapsa sulfexigens* (OTU 25758), in addition to SOB, specifically proliferated at 2–10 mm, a depth at which S^0^ had highly accumulated (Table 4). The sulfur disproportionator transformed S^0^ to sulfide and sulfate, resulting in a resupply of the electron donor to SOB. Thus, the sulfur disproportionator also played an important role in the sulfur cycle in the interface layer, which was consistent with previous findings demonstrating the involvement of sulfur disproportionators in natural sulfur cycling (9, 10, 31).

On the other hand, the two different SRB, *i.e*., *Desulfotignum toluenicum* (OTU 20456) and *Desulfosalsimonas propionicica* (OTU 20168), specifically prevailed at 2–10 mm, a depth at which they showed more than ten-fold higher relative abundances than at the other depths (Table 4). Chemolithotrophs play an important role in the carbon cycle under dark conditions as the major primary producers (22, 23). Thus, carbon fixed by chemolithotrophic SOB and the sulfur-disproportionating bacterium appeared to serve as the carbon source of these chemoorganotrophic SRB, thereby facilitating the degradation of organic matter in the oxic-anoxic boundary layer of the sediment.

Although no SOB were predominant at 0–2 mm (Table 3), the concentration of sulfate ions was higher than at the deeper depths (Table 2). Our previous laboratory-scale incubation of the launched sediment indicated the rapid increase and decrease in SOB coincided with the accumulation of sulfate ions at 0–2 mm (16). Thus, the high concentration of sulfate ions found in the present study may reflect SOB activity in the uppermost layer. In the anoxic layers at a depth of 10–40 mm, deltaproteobacterial SRB prevailed, which is consistent with our previous finding (16). Sulfur disproportionating bacteria, including *Desulfocapsa sulfexigens* (OTU 25758), did not dominate outside the oxic-anoxic interface, indicating the negligible contribution of these bacteria to sulfur transformation at the surface and in deeper layers.

Due to the difficulties associated with analyzing reduced inorganic sulfur species in the limited amounts of vertical sections of the launched sediment sample, the identity and distribution of the sulfur species in the oxic-anoxic boundary layer remained unclear. Using the new sulfur-fractionation method, we successfully identified the inorganic sulfur species in the sediment. In parallel with geochemical profiling, the vertical localization of intrinsic microorganisms was analyzed in detail by the high-throughput Illumina sequencing of 16S rRNA genes, providing important insights into microbial sulfur cycling in the interface layer. The present results strongly suggest that SOB, a sulfur-disproportionating bacterium, and SRB were involved in sulfur cycling with S^0^ as a pivotal sulfur species in the oxic-anoxic boundary layer of the launched marine sediment.

## Supplementary information



## Figures and Tables

**Table 1:** Physicochemical properties of the launched sediment.

Depth (mm)	ORP (mV)^a^	Ignition Loss (%)^b^	Concentration (g kg^−1^ dry sediment)^a^		

			Cl^−^	Total carbon (TC)	Total nitrogen (TN)
0–2	161±30.1	15.8±0.02	26.2±0.51	49.5±3.0	5.1±0.4
2–10	−58±9.6	11.8±0.08	20.0±1.89	33.4±0.0	2.7±0.0
10–20	−106±7.5	11.8±0.52	24.1±0.28	34.5±1.1	2.7±0.0

a Measurements were conducted in triplicate. “±” indicates the standard deviation of three replications

b Measurements were conducted in duplicate. “±” indicates the variation between two replications

**Table 2:** Concentrations of inorganic sulfur species and TS in the sediment.

Depth (mm)	Concentration (gS kg^−1^ dry sediment)^a^				

	SO_4_^2−^	AVS	CRS	ES^b^	TS
0–2	1.71±0.04	0.23±0.29	0.33±0.10	0.37±0.04	7.25±0.99
2–10	1.24±0.11	2.70±0.59	6.29±3.10	3.60±0.53	12.04±0.14
10–20	1.37±0.01	3.09±1.30	9.38±3.18	1.84±0.68	13.49±0.17

a “±” indicates the standard deviation of three replications

b The concentration of ES at 2–10 mm was significantly higher than those at the other depths (*P*<0.05)

**Table 3:** Most abundant OTUs and their closest relatives found in the sediment.

Depth (mm)	OTU No.	Closest relative^a^	Identity (%)	Accession No.	Phylum/Class	Relative abundance (%)^b^	Inorganic sulfur transformation^c^	L/P/O^d^
0–2	21521	*Marinobacter salicampi*	100	EF486354	*Gammaproteobacteria*	6.0±0.8	N.A.	O
	22221	*Gaetbulibacter aestuarii*	100	GU552681	*Bacteroidetes*	5.2±0.2	N.A.	O
	24312	*Schizothrix calcicola*	100	AY274615	*Cyanobacteria*	4.7±2.2	N.A.	P
	24314	*Marinobacter salsuginis*	100	MG575733	*Gammaproteobacteria*	3.4±0.5	N.A.	O
	19166	*Puniceibacterium antarcticum*	100	JX070673	*Alphaproteobacteria*	3.3±0.5	N.A.	O

2–10	15133	*Sulfurimonas denitrificans*	94	L40808	*Epsilonproteobacteria*	24.8±1.3	SO	L
	25758	*Desulfocapsa sulfexigens*	97	Y13672	*Deltaproteobacteria*	7.1±2.0	SD	L
	7171	*Halanaerobium acetethylicum*	88	U32594	*Firmicutes*	3.0±0.2	N.A.	O
	15113	*Alkaliphilus oremlandii*	88	MG264216	*Firmicutes*	2.4±0.4	N.A.	O
	4080	*Sulfurimonas autotrophica*	90	CP002205	*Epsilonproteobacteria*	2.3±0.6	SO	L

10–20	6683	*Thermomarinilinea lacunifontana*	88	AB669272	*Chloroflexi*	4.0±0.7	N.A.	O
	1686	*Desulfobulbus elongatus*	92	X95180	*Deltaproteobacteria*	2.8±0.4	SR	O
	15642	*Desulfuromonas svalbardensis*	94	AY835390	*Deltaproteobacteria*	2.0±0.1	S^0^R	O
	25436	*Malonomonas rubra*	95	Y17712	*Deltaproteobacteria*	1.7±0.1	N.A.	O
	18095	*Desulfofaba fastidiosa*	95	AY268891	*Deltaproteobacteria*	1.5±0.1	SR	O

20–40	1686	*Desulfobulbus elongatus*	92	X95180	*Deltaproteobacteria*	2.7±0.1	SR	O
	15642	*Desulfuromonas svalbardensis*	94	AY835390	*Deltaproteobacteria*	2.6±0.5	S^0^R	O
	6683	*Thermomarinilinea lacunifontana*	88	AB669272	*Chloroflexi*	2.4±0.3	N.A.	O
	25436	*Malonomonas rubra*	95	Y17712	*Deltaproteobacteria*	1.8±0.1	N.A.	O
	19673	*Methylobacter marinus*	100	LT220841	*Gammaproteobacteria*	1.8±0.5	N.A.	O

a The closest relatives were assigned on BLAST in the DDBJ

b “±” indicates the standard deviation of three replications

c The inorganic sulfur transformation ability of the closest relative: SO, sulfur oxidation; SD, sulfur disproportionation; SR, sulfate reduction; S^0^R, sulfur reduction. N.A. indicates not applicable

d “L” indicates chemolithotroph, “P” indicates photolithotroph, and “O” indicates chemoorganotroph

**Table 4:** Relative abundance of OTU with a high increasing ratio at 2–10 mm^a^.

OTU No.	Closest relative^b^	Identity (%)	Accession No.	Class	Relative abundance (%)^c^				Inorganic sulfur transformation^d^	L/O^e^

					0–2 mm	2–10 mm	10–20 mm	20–40 mm		
15133	*Sulfurimonas denitrificans*	94	L40808	*Epsilonproteobacteria*	1.99±0.38	24.8±1.28	0.64±0.17	0.37±0.06	SO	L
25758	*Desulfocapsa sulfexigens*	97	Y13672	*Deltaproteobacteria*	0.13±0.02	7.07±1.98	0.31±0.18	0.09±0.02	SD	L
4080	*Sulfurimonas autotrophica*	90	CP002205	*Epsilonproteobacteria*	0.14±0.09	2.31±0.55	0.03±0.01	0.03±0.01	SO	L
20456	*Desulfotignum toluenicum*	100	MG264282	*Deltaproteobacteria*	0.14±0.03	1.76±0.51	0.17±0.11	N.D.	SR	O
4041	*Mariprofundus micogutta*	97	LC107871	*Zetaproteobacteria*	0.01±0.01	0.16±0.04	N.D.	N.D.	N.A.	L
20168	*Desulfosalsimonas propionicica*	99	DQ067422	*Deltaproteobacteria*	0.01±0.01	0.13±0.01	0.01±0.01	N.D.	SR	O

a The OTUs showing more than a ten-fold higher relative abundance at 2–10 mm than at the other depths

b The closest relatives were assigned on BLAST in the DDBJ

c “±” indicates the standard deviation of three replications. N.D. indicates not detected

d The inorganic sulfur transformation ability of the closest relative: SO, sulfur oxidation; SD, sulfur disproportionation; SR, sulfate reduction. N.A. indicates not applicable

e “L” indicates chemolithotroph and “O” indicates chemoorganotroph

## References

[1] Altschul S.F., Madden T.L., Schäffer A.A., Zhang J., Zhang Z., Miller W., Lipman D.J. (1997). Gapped BLAST and PSI-BLAST: a new generation of protein database search programs. Nucl Acids Res.

[2] Aoyagi T., Kimura M., Yamada N. (2015). Dynamic transition of chemolithotrophic sulfur-oxidizing bacteria in response to amendment with nitrate in deposited marine sediments. Front Microbiol.

[3] Bacosa H.P., Inoue C. (2015). Polycyclic aromatic hydrocarbons (PAHs) biodegradation potential and diversity of microbial consortia enriched from tsunami sediments in Miyagi, Japan. J Hazard Mater.

[4] Burton E.D., Sullivan L.A., Bush R.T., Johnston S.G., Keene A.F. (2008). A simple and inexpensive chromium-reducible sulfur method for acid-sulfate soils. Appl Geochem.

[5] Canfield D.E., Raiswell R., Westrich J.T., Reaves C.M., Berner R.A. (1986). The use of chromium reduction in the analysis of reduced inorganic sulfur in sediments and shales. Chem Geol.

[6] Caporaso J.G., Kuczynski J., Stombaugh J. (2010). QIIME allows analysis of high-throughput community sequencing data. Nat Methods.

[7] Caporaso J.G., Lauber C.L., Walters W.A. (2011). Global patterns of 16S rRNA diversity at a depth of millions of sequences per sample. Proc Natl Acad Sci USA.

[8] Caporaso J.G., Lauber C.L., Walters W.A. (2012). Ultra-high-throughput microbial community analysis on the Illumina HiSeq and MiSeq platforms. ISME J.

[9] Elshahed M.S., Senko J.M., Najar F.Z., Kenton S.M., Roe B.A., Dewers T.A., Spear J.R., Krumholz L.R. (2003). Bacterial diversity and sulfur cycling in a mesophilic sulfide-rich spring. Appl Environ Microbiol.

[10] Finster K., Liesack W., Thamdrup B.O. (1998). Elemental sulfur and thiosulfate disproportionation by *Desulfocapsa sulfoexigens* sp. nov., a new anaerobic bacterium isolated from marine surface sediment. Appl Environ Microbiol.

[11] Hamilton T.L., Jones D.S., Schaperdoth I., Macalady J.L. (2015). Metagenomic insights into S(0) precipitation in a terrestrial subsurface lithoautotrophic ecosystem. Front Microbiol.

[12] Han Y., Perner M. (2015). The globally widespread genus *Sulfurimonas*: versatile energy metabolisms and adaptations to redox clines. Front Microbiol.

[13] Hori T., Kimura M., Aoyagi T., Navarro R.R., Ogata A., Sakoda A., Katayama Y., Takasaki M. (2014). Biodegradation potential of organically enriched sediments under sulfate- and iron-reducing conditions as revealed by the 16S rRNA deep sequencing. J Water Environ Technol.

[14] Hsieh Y.P., Yang C.H. (1989). Diffusion methods for the determination of reduced inorganic sulfur species in sediments. Limnol Oceanogr.

[15] Hsieh Y.P., Shieh Y.N. (1997). Analysis of reduced inorganic sulfur by diffusion methods: improved apparatus and evaluation for sulfur isotopic studies. Chem Geol.

[16] Ihara H., Hori T., Aoyagi T., Takasaki M., Katayama Y. (2017). Sulfur-oxidizing bacteria mediate microbial community succession and element cycling in launched marine sediment. Front Microbiol.

[17] Itoh H., Aita M., Nagayama A., Meng X.Y., Kamagata Y., Navarro R., Hori T., Ohgiya S., Kikuchi Y. (2014). Evidence of environmental and vertical transmission of *Burkholderia* symbionts in the oriental chinch bug, *Cavelerius saccharivorus* (Heteroptera: Blissidae). Appl Environ Microbiol.

[18] Jørgensen B.B. (1977). The sulfur cycle of a coastal marine sediment (Limfjorden Denmark). Limnol Oceanogr.

[19] Lavik G., Stührmann T., Brüchert V. (2009). Detoxification of sulphidic African shelf waters by blooming chemolithotrophs. Nature.

[20] Makita K., Inoshita K., Kayano T. (2014). Temporal changes in environmental health risks and socio-psychological status in areas affected by the 2011 tsunami in Ishinomaki, Japan. Environ Pollut.

[21] Manheim F.T. (1974). Comparative studies on extraction of sediment interstitial waters: Discussion and comment on the current state of interstitial water studies. Clays Clay Miner.

[22] Mattes T.E., Nunn B.L., Marshall K.T., Proskurowski G., Kelley D.S., Kawka O.E., Goodlett D.R., Hansell D.A., Morris R.M. (2013). Sulfur oxidizers dominate carbon fixation at a biogeochemical hot spot in the dark ocean. ISME J.

[23] Middelburg J.J. (2011). Chemoautotrophy in the ocean. Geophys Res Lett.

[24] Mimura N., Yasuhara K., Kawagoe S., Yokoki H., Kazama S. (2011). Damage from the Great East Japan Earthquake and Tsunami—a quick report. Mitig Adapt Strateg Glob Chang.

[25] Nakamura K., Kuwatani T., Kawabe Y., Komai T. (2016). Extraction of heavy metals characteristics of the 2011 Tohoku tsunami deposits using multiple classification analysis. Chemosphere.

[26] Okabe S., Ito T., Sugita K., Satoh H. (2005). Succession of internal sulfur cycles and sulfur-oxidizing bacterial communities in microaerophilic wastewater biofilms. Appl Environ Microbiol.

[27] Pjevac P., Kamyshny A., Dyksma S., Mußmann M. (2014). Microbial consumption of zero-valence sulfur in marine benthic habitats. Environ Microbiol.

[28] Rohwerder T., Sand W. (2003). The sulfane sulfur of persulfides is the actual substrate of the sulfur-oxidizing enzymes from *Acidithiobacillus* and *Acidiphilium* spp. Microbiology.

[29] Sera K., Goto S., Takahashi C., Saitoh Y., Yamauchi K. (2014). Effects of heavy elements in the sludge conveyed by the 2011 tsunami on human health and the recovery of the marine ecosystem. Nucl Instrum Methods Phys Res, Sect B.

[30] Sievert S.M., Scott K.M., Klotz M.G. (2008). Genome of the epsilonproteobacterial chemolithoautotroph *Sulfurimonas denitrificans*. Appl Environ Microbiol.

[31] Thamdrup B.O., Finster K., Hansen J., Bak F. (1993). Bacterial disproportionation of elemental sulfur coupled to chemical reduction of iron or manganese. Appl Environ Microbiol.

[32] van den Ende F.P., van Gemerden H. (1993). Sulfide oxidation under oxygen limitation by a *Thiobacillus thioparus* isolated from a marine microbial mat. FEMS Microbiol Ecol.

[33] Wieder R.K., Lang G.E., Granus V.A. (1985). An evaluation of wet chemical methods for quantifying sulfur fractions in freshwater wetland peat. Limnol Oceanogr.

[34] Yamamoto M., Takai K. (2011). Sulfur metabolisms in epsilon-and gamma-Proteobacteria in deep-sea hydrothermal fields. Front Microbiol.

