# Supplementary material for: Stratification of Sulfur Species and Microbial Community in Launched Marine Sediment by an Improved Sulfur-Fractionation Method and 16S rRNA Gene Sequencing
Source: Microbes Environ. 2019 Jun 11;34(2):199–205. doi: 10.1264/jsme2.ME18153 (PMC6594742; doi:10.1264/jsme2.ME18153)
Supplement: Supplementary file 1 [file 34_199_s1.pdf]

Supplementary information

Stratification of Sulfur Species and Microbial Community in Launched Marine Sediment by an Improved Sulfur-Fractionation Method and 16S rRNA Gene Sequencing

Hideyuki Ihara, Tomoyuki Hori*, Tomo Aoyagi, Hiroki Hosono, Mitsuru Takasaki, Yoko Katayama*

***Correspondence**: Tomoyuki Hori[1)], Yoko Katayama[2)]

[1)] Phone/Fax: +81 29 861 8326, e-mail: hori-tomo@aist.go.jp.

[2)] Phone: +81 3 3823 2408, Fax:+81 3 3822 3247,
e-mail: katayama@cc.tuat.ac.jp.

Table S1 Recovery efficiency of FeS, FeS_2 and S^0 in the improved sulfur fractionation method

Substance	Sulfur subjected (mgS)	Sulfur recovered (mgS)	Recovery (%)	Average recovery (%)
FeS [a]	0.320	0.315	98.5	94.9 ± 2.7
	0.235	0.221	94.0	
	0.221	0.203	92.0	
	0.119	0.113	94.9	
FeS_2 [b]	0.365	0.367	100.3	97.8 ± 2.7
	0.445	0.423	95.0	
	0.468	0.460	98.2	
S^0 [c]	0.123	0.117	95.1	99.9 ± 5.3
	0.062	0.061	99.0	
	0.123	0.130	105.6	

[a] FeS was reacted with 9 M HCl to recover the AVS fraction

[b] FeS_2 was reacted with 9 M HCl, and then 2 M Cr(II) to recover the CRS fraction

[c] S^0 was reacted with 9 M HCl, 2 M Cr(II), and then 99.5% N,N-dimetylformamide to recover the ES fraction

Table S2 Summary of high-throughput Illumina sequencing of 16S rRNA genes from the launched sediment [a]

Depth (mm)	No. of sequence	No. of OTUs	α-diversity indices [b]		
			Chao1	Shannon	1/Simpson
0-2	45134 ± 7152	1818 ± 212	3485 ± 709	7.5 ± 0.2	59.0 ± 4.6
2-10	40709 ± 3467	4923 ± 200	10443 ± 522	7.8 ± 0.1	16.0 ± 0.9
10-20	45518 ± 2676	7132 ± 990	13850 ± 236	10.4 ± 0.0	232 ± 23
20-40	41330 ± 2113	6971 ± 810	14024 ± 374	10.3 ± 0.0	238 ± 4

[a] "±" indicates the standard deviation of three replications.

[b] Diversity indices were calculated by using an equal number of sequences (*n*=37762) subsampled 10 times.

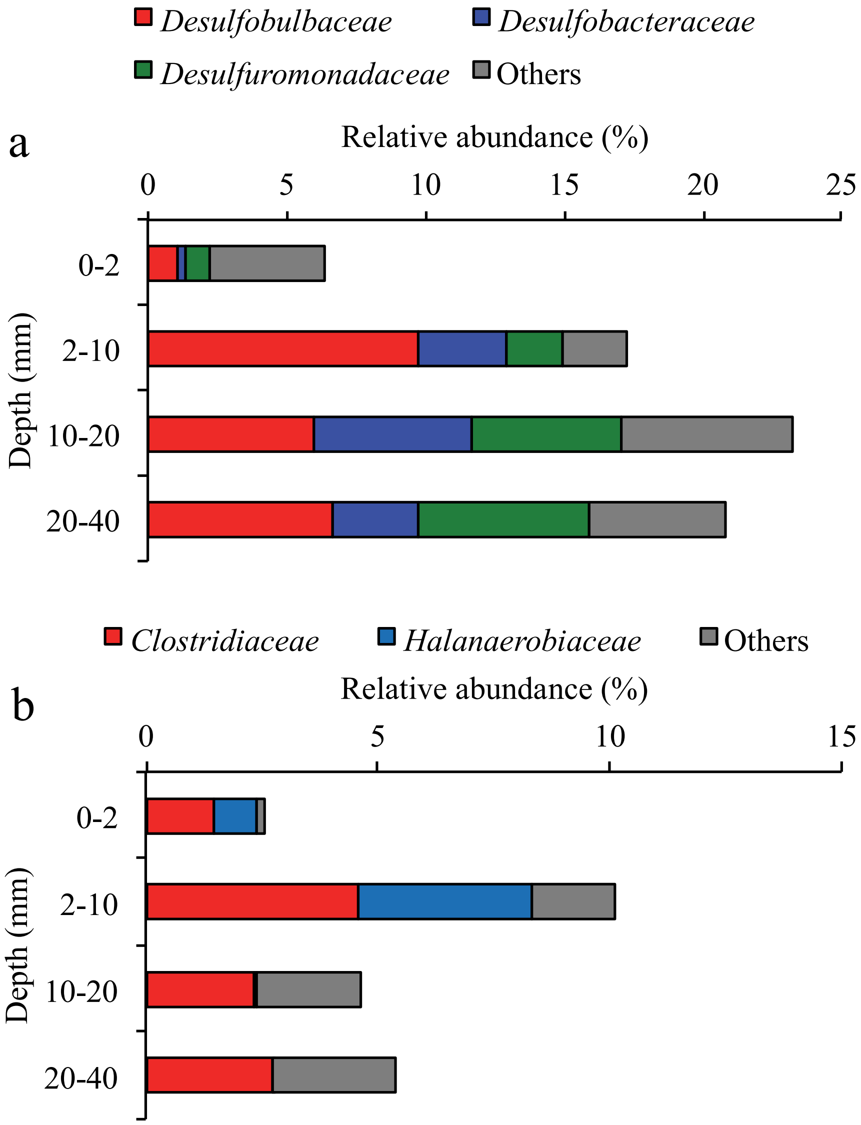

Fig. S1 Vertical changes in relative abundances of OTUs indicated at the taxonomic level of family in the class Deltaproteobacteria (a) and Clostridia (b). The relative abundances are averages of three replications

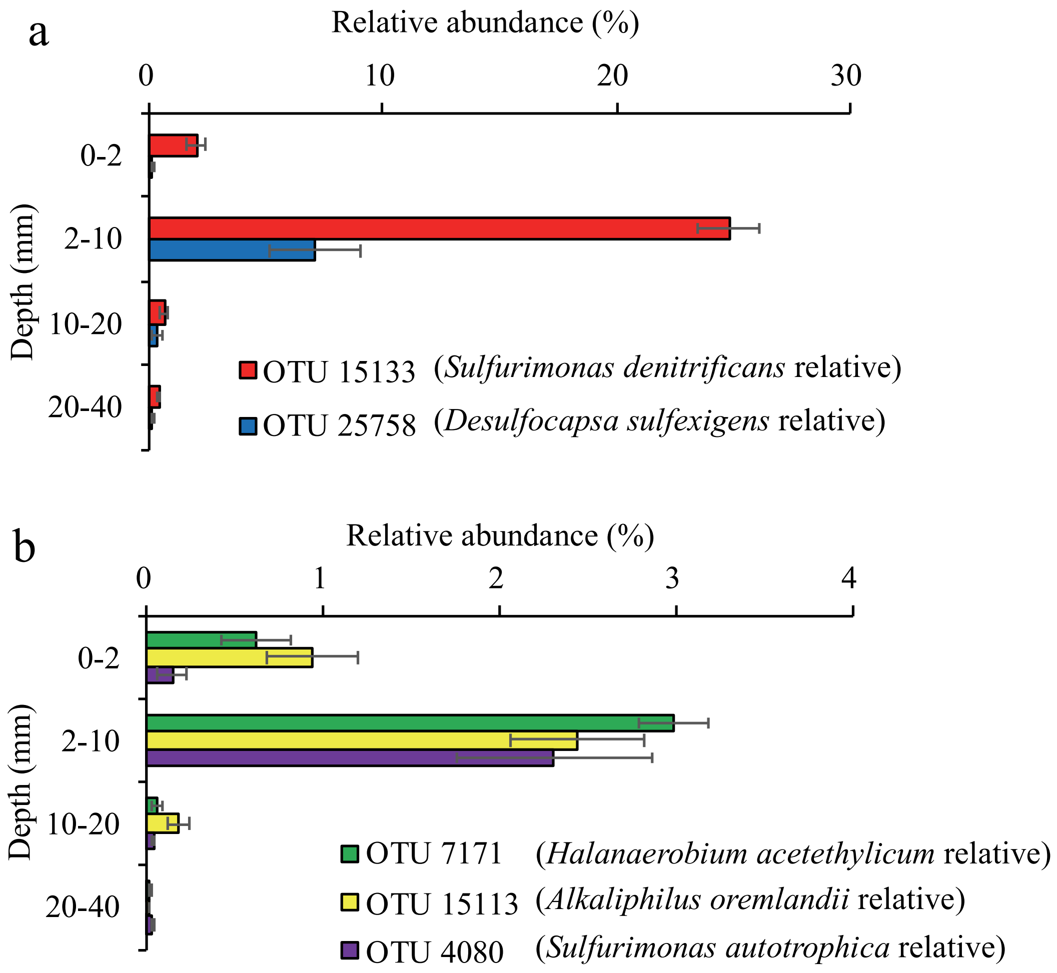

Fig. S2 Vertical changes in relative abundances of the five most abundant OTUs in the 2-10 mm depth layer. (a), OTUs 15133 and 25758; (b), OTUs 7171, 15113 and 4080. The relative abundances are averages of three replications and error bars indicate the standard deviations
